# Non-dipping pattern of nocturnal blood pressure as a risk factor for macular ischemia in branch retinal vein occlusion

**DOI:** 10.1038/s41598-021-89915-9

**Published:** 2021-05-18

**Authors:** Gwang Myeong Noh, Haeyoung Lee, Hyun Duck Kwak, Hyun Wong Kim, Sang Joon Lee

**Affiliations:** 1 Department of Ophthalmology, Kosin University College of Medicine, #34 Amman-dong, Suh-ku, Busan, 602-702 South Korea; 2 Department of Thoracic and Cardiovascular Surgery, Kosin University College of Medicine, Busan, South Korea; 3 Inje University College of Medicine, Busan, South Korea

**Keywords:** Risk factors, Cardiovascular diseases, Eye diseases

## Abstract

Branch retinal vein occlusion (BRVO) is ocular vascular disease affecting approximately 14 million people worldwide, and is closely associated with high blood pressure (BP). Although macular ischemia is a critical factor in the visual prognosis of BRVO, the relationship between macular ischemia and different patterns of nocturnal BP is unknown. Here, we investigated whether a dipping pattern of nocturnal BP is associated with the development of macular ischemia in patients with BRVO. A total of 273 patients were reviewed; of these, 86 (86 eyes) patients were included. All recruited patients had a macular thickness map by optical coherence tomography and underwent 24-h ambulatory BP monitoring. According to their dipping patterns, the participants were divided into dipper and non-dipper groups. The non-dipper group had worse visual outcomes at the initial and 6-month visits (*P* = 0.014 and *P* = 0.003, respectively). Five of 32 eyes (15.6%) in the dipper group and 32 of 54 (59.3%) in the non-dipper group had macular ischemia. In a multivariate analysis, the night-to-day systolic BP ratio was associated with the degree of macular ischemia (β = − 0.313, *P* = 0.004). Thus, a non-dipping pattern may be a risk factor for macular ischemia in patients with BRVO.

## Introduction

Retinal vein occlusion (RVO) is an ocular vascular disease affecting approximately 16 million people worldwide^1^. The prevalence of branch retinal vein occlusion (BRVO) in patients with RVO is 4.42/1000 adults, an estimated 13.9 million adults worldwide^2^. It is the second most common vision-threatening retinal vascular disease^3^. The visual outcomes of patients with BRVO depend on their macular condition, which is affected by impaired blood circulation, such as macular edema and ischemia^4–6^. In particular, macular ischemia has been identified as one of factors for visual outcomes^4–6^.

Ischemia in macular area can damage the integrity of the foveal photoreceptor which was correlated with visual outcomes in the patients of BRVO^4–6^. The damage of photoreceptor corresponded to area of capillary nonperfusion at the level of the deep capillary plexus can be detected by optical coherence tomography angiography^6^. In this background, macular ischemia is the most critical factor in determining the visual prognosis of BRVO^5–8^. However, it is not well known which factors contribute to the development of macular ischemia in patients with BRVO.

Systemic vascular diseases such as hypertension and arteriosclerosis are risk factors for BRVO^2,9^. Specifically, hypertension is closely associated with macular edema in patients with RVO^10^. It has been reported that successful treatment of systemic hypertension alone could be sufficient to resolve macular edema^10^. The reduction of macular edema following systemic hypertension treatment suggests that systemic vascular conditions are key factors for retinal vascular diseases^10,11^. In addition to the degree of blood pressure (BP), the patterns of circadian BP variation have been implicated in targeted organ damage, including cerebral blood vessels and cardiovascular systems^12^. The patterns of systolic BP can be classified according to the systolic night-day BP ratio. Clinical terms such as “dipper,” “non-dipper,” and “riser” have evolved to include people whose BP declines by over 10% during nighttime, those whose BP declines by less than 10% during nighttime, and those who have a higher BP during nighttime compared to that in daytime, respectively^12^. For assessing the patterns of BP, 24-h ambulatory blood pressure monitoring (ABPM), as opposed to clinical BP monitoring, is highly useful. The pattern of 24-h ABPM is associated with microalbuminuria and early diabetic nephropathy and is a key predictor of cardiovascular outcomes^13–16^.

In the ophthalmic field, several studies have investigated the patterns of ABPM in patients with BRVO^17–19^. In a previous study, a higher prevalence of hypertension was observed in patients with BRVO, and approximately half of the patients with hypertension were non-dippers^19^. RVO studies in North America have suggested that 24-h ABPM is a useful tool for detecting uncontrolled nocturnal hypertension, and patients with RVO have a higher prevalence of non-dipping patterns^17,18^. These studies have reported that nocturnal hypertension is a risk factor for BRVO. However, to the best of our knowledge, the effects of nocturnal BP pattern on the development of macular ischemia in patients with BRVO have not been investigated yet. Hence, we investigated the association between nocturnal BP patterns and the development of macular ischemia in patients with BRVO.

## Results

### Clinical characteristics

A total of 273 patients were reviewed, and 86 patients (86 eyes) were included after the exclusion criteria were applied. These patients were followed up for a minimum of 12 (mean, 13 ± 1; range, 12–14) months.

The clinical characteristics of the dipper and non-dipper groups are summarized in Table 1. Of the 86 eyes, 32 (37.2%) belonged to the dipper group and 54 (62.8%) belonged to the non-dipper group. The mean age of the patients with BRVO was 59.2 ± 1.7 years in the dipper group and 61.2 ± 1.6 years in the non-dipper group, demonstrating no significant difference between the two groups (*p* = 0.409). There were no significant differences in the distribution of right/left eye affected, follow-up time, baseline intraocular pressure (IOP), mean counts of anti-vascular endothelial growth factor (VEGF) injection and laser treatment sessions, axial length, smoking history, and alcohol history between the two groups.

**Table 1:** Clinical characteristics of the dipper and non-dipper groups of patients diagnosed with branch retinal vein occlusion according to the dipping pattern of the nocturnal systolic blood pressure.

Variables	Dipper (n = 32)	Non-dipper (n = 54)	*p*-value
Mean age (years)	59.2 ± 1.7	61.2 ± 1.6	0.409^b^
Male/female (%)	23/9 (71.9, 28.1)	24/30 (44.4, 55.6)	0.393^a^
Affected eyes (right/left) (%)	19/13 (59.4, 40.6)	29/25 (53.7, 46.3)	0.886^a^
Follow-up time (months)	13 ± 2	13 ± 1	0.908^b^
Baseline mean IOP (mmHg)	14 ± 0.6	15 ± 0.4	0.545^b^
Mean count of anti-VEGF injections	2.4 ± 0.5	2.0 ± 0.3	0.559^b^
Mean count of laser treatment sessions	1.1 ± 0.3	0.7 ± 0.2	0.173^b^
AL in affected eye (mm)	23.8 ± 0.4	23.4 ± 0.8	0.374^b^
AL in unaffected eye (mm)	24.0 ± 1.2	23.6 ± 0.8	0.224^b^
**Medication history**			
Diabetes mellitus (%)	0 (0)	0 (0)	–
Hypertension (%)	0 (0)	0 (0)	–
Smoking history (%)	4 (12.5)	8 (14.8)	0.784^a^
Alcohol history (%)	6 (18.8)	9 (16.7)	0.322^a^
24 h ambulatory mean SBP (mmHg)	143.2 ± 2.9	138.2 ± 2.2	0.163^b^
24 h ambulatory mean DBP (mmHg)	89.5 ± 2.1	86.0 ± 1.4	0.145^b^
Daytime SBP, mean (mmHg)	149.3 ± 2.9	138.8 ± 2.3	**0.006**^b^
Daytime DBP, mean (mmHg)	93.2 ± 2.0	86.4 ± 1.5	**0.008**^b^
Nighttime SBP, mean (mmHg)	127.3 ± 3.0	134.4 ± 2.1	**0.048**^b^
Nighttime DBP, mean (mmHg)	80.0 ± 2.3	83.2 ± 1.4	0.208^b^
Night-to-day SBP ratio, mean (%)	14.7 ± 0.9	3.0 ± 0.8	** < 0.0001**^b^

Data are presented as mean ± standard deviation or number (%).

Night to day systolic blood pressure ratio (%) = (Daytime sysBP − Nighttime sysBP)/Daytime sysBP × 100.

Dipper ≥ 10%; Non-dipper < 10%.

Statistically significant values appear in boldface.

*IOP* intraocular pressure, *VEGF* vascular endothelial growth factor, *AL* axial length.

^a^Based on Pearson’s chi-square test for categorical variables.

^b^Based on Student’s t-test for continuous variables.

### Comparative analysis of 24-h ambulatory blood pressure monitoring

The results of the 24-h ABPM characteristics between the dipper and non-dipper groups are shown in Table 1. The 24-h mean systolic and diastolic BP showed no significant differences between the two groups (*p* = 0.163 and *p* = 0.145, respectively). The daytime systolic and diastolic BPs in the dipper group were 149.3 ± 2.9 mmHg and 93.2 ± 2.0 mmHg, respectively, which were significantly higher than those of the non-dipper group, 138.8 ± 2.3 mmHg and 86.4 ± 1.5 mmHg, respectively (*p* = 0.006). The nighttime systolic BP in the non-dipper group, 134.4 ± 2.1 mmHg, was significantly higher than that in the dipper group, which was 127.3 ± 3.0 mmHg (*p* = 0.048). The night-to-day systolic BP ratios in the dipper and non-dipper groups were 14.7 ± 0.9% and 3.0 ± 0.8%, respectively (*p* < 0.0001).

### Central macular thickness, choroidal thickness, and macular ischemia

**Figure 1:**
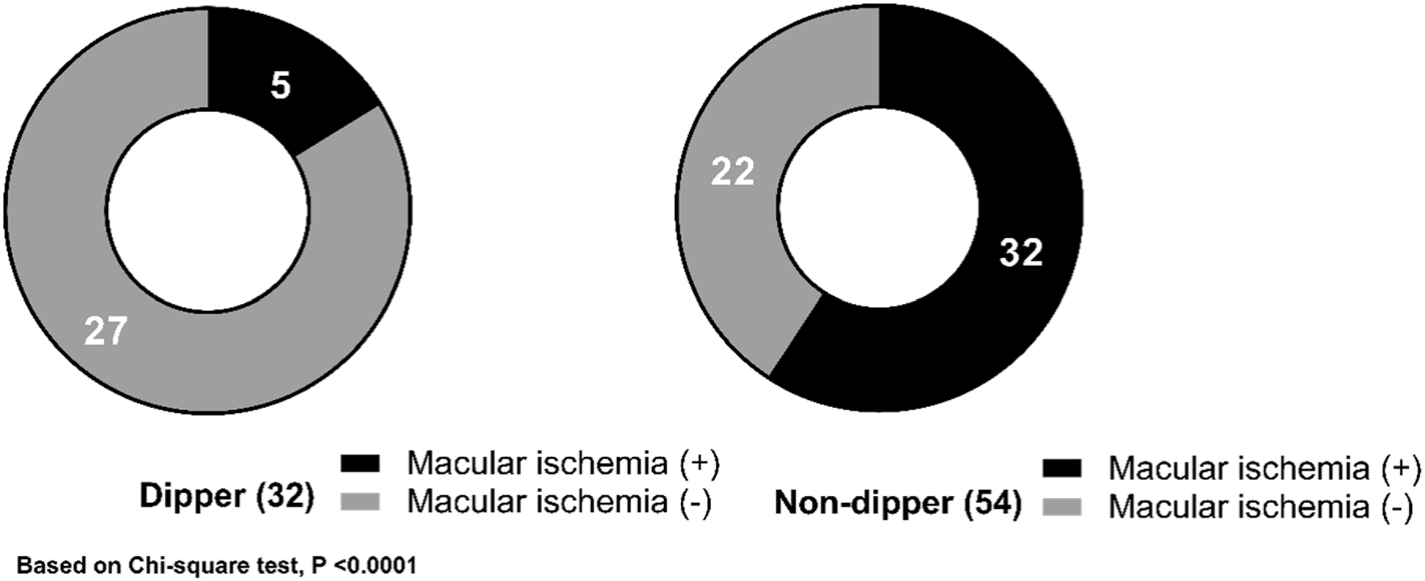
The proportion of macular ischemia is compared between the dipper and non-dipper groups. The non-dipper group demonstrates a high prevalence of macular ischemia.

The central macular thickness (CMT), choroidal thickness, and grade of macular ischemia of the dipper and non-dipper groups are listed in Table 2. The CMT at baseline was thicker in the non-dipper group (416 ± 24 µm in the non-dipper group vs. 341 ± 19 µm in the dipper group, *p* = 0.017). However, the difference in baseline CMT between the two groups disappeared after 6 months (283 ± 13 µm vs. 289 ± 12 µm, *p* = 0.744). The extent of change in CMT from baseline to after 6 months was found to be significantly greater in the non-dipper group (141 ± 26 µm vs. 50 ± 20 µm, *p* = 0.007). A comparison of subfoveal choroidal thickness (SFCT) revealed no significant difference between the two groups (*p* = 0.737). The degree of macular ischemia between the two groups was significantly different (*p* = 0.001). There was macular ischemia in 15.6% and 59.3% of the dipper and non-dipper groups, respectively (Fig. 1).

**Table 2:** Comparison of central macular thickness, choroidal thickness, and macular ischemia between the dipper and non-dipper groups of patients diagnosed with branch retinal vein occlusion.

Variable	Dipper (n = 32)	Non-dipper (n = 54)	*p*-value
Baseline CMT in affected eye (µm)	341 ± 19	416 ± 24	**0.017**^a^
Baseline CMT in unaffected eye (µm)	265 ± 3	255 ± 5	0.134^a^
> 6 mos. CMT in affected eye (µm)	289 ± 12	283 ± 13	0.744^a^
> 6 mos. CMT in unaffected eye (µm)	268 ± 3	265 ± 5	0.687^a^
CMT changes between initial and > 6 mos. (µm)	50 ± 20	141 ± 26	**0.007**^a^
Baseline SFCT in affected eye (µm)	306 ± 28	296 ± 16	0.737^a^
Baseline SFCT in unaffected eye (µm)	224 ± 31	262 ± 18	0.268^a^
**Macular ischemia grade**			**0.001**^b^
0 quadrant	27	22	
1 quadrant	3	11	
2 quadrant	1	15	
3 quadrant	1	6	

Data are presented as mean ± standard deviation.

Night to day systolic blood pressure ratio (%) = (Daytime sysBP − Nighttime sysBP)/Daytime sysBP × 100.

Dipper ≥ 10%; Non-dipper < 10%.

Statistically significant values appear in boldface.

*CMT* central macular thickness, *mos.* months, *SFCT* subfoveal choroidal thickness.

^a^Based-on Student’s t-test.

^b^Based-on Chi-square.

### Characteristics of visual outcome

The mean visual acuity of the two groups from baseline to 6 months is shown in Table 3. The visual outcome of the non-dipper group was worse than that of the dipper group at both the baseline and 6-month visits (*p* = 0.014 and *p* = 0.003, respectively). However, the changes in visual acuity between the initial and final visits were not significantly different between the two groups (*P* = 0.364). Significant differences between the two groups at both the baseline and 6-month visits were also maintained in the multivariate linear regression analysis. (*p* = 0.019 and *p* = 0.036, respectively).

**Table 3:** Multivariate linear regression analysis of visual acuity between the dipper and non-dipper groups of patients with branch retinal vein occlusion.

Variable	Dipper (n = 32)	Non-dipper (n = 54)	*p*-value
**Baseline logMAR BCVA (Snellen)**			
Crude	0.33 ± 0.06 (20/43)	0.53 ± 0.05 (20/68)	**0.014**
Adjusted	0.33 ± 0.05 (20/43)	0.51 ± 0.04 (20/65)	**0.019**
** > 6 mos. logMAR BCVA (Snellen)**			
Crude	0.16 ± 0.06 (20/29)	0.39 ± 0.05 (20/49)	**0.003**
Adjusted	0.22 ± 0.05 (20/33)	0.37 ± 0.04 (20/46)	**0.036**
**LogMAR BCVA changes between initial and > 6 mos. (Snellen)**			
Crude	0.17 ± 0.06 (20/30)	0.11 ± 0.03 (20/26)	0.364
Adjusted	0.15 ± 0.05 (20/28)	0.10 ± 0.04 (20/25)	0.433

The non-dipper group showed significantly worse visual outcomes when compared to the dipper group.

Night to day systolic blood pressure ratio (%) = (Daytime sysBP − Nighttime sysBP)/Daytime sysBP × 100.

Dipper ≥ 10%; Non-dipper < 10%.

Statistically significant values appear in boldface.

Adjusted for variables in basic and clinical parameters associated with visual acuity (age, sex, body mass index, current smoking status, and alcohol drinking status).

*BCVA* best-corrected visual acuity, *logMAR* logarithm of minimum angle resolution.

### Univariate analysis

Table 4 presents the results of a univariate analysis of the association between the degree of macular ischemia and 24-h ABPM parameters. The night-to-day systolic BP ratio was significantly associated with the degree of macular ischemia (β = − 0.320, *P* = 0.003). However, the 24-h mean systolic and diastolic BP (β = − 0.143, *P* = 0.189 and β = − 0.155, *P* = 0.155, respectively), daytime mean systolic and diastolic BP (β = − 0.169, *P* = 0.120 and β = − 0.195, *P* = 0.073, respectively), and nighttime mean systolic and diastolic BP (β = 0.041, *P* = 0.711 and β = 0.046, *P* = 0.671, respectively) showed no association with macular ischemia.

**Table 4:** Univariable and multivariable linear regression analyses of the association between degree of macular ischemia and blood pressure in dipper and non-dipper group.

Variable	Unadjusted analysis		Adjusted analysis	
	β	*p* value^a^	β	*p* value^a^
24 h mean SBP (mmHg)	− 0.143	0.189	− 0.136	0.217
24 h mean DBP (mmHg)	− 0.155	0.155	− 0.123	0.289
Daytime SBP, mean (mmHg)	− 0.169	0.120	− 0.157	0.158
Daytime DBP, mean (mmHg)	− 0.195	0.073	− 0.165	0.160
Nighttime SBP, mean (mmHg)	0.041	0.711	0.049	0.660
Nighttime DBP, mean (mmHg)	0.046	0.671	0.101	0.383
Night-to-day SBP ratio, mean (%)	− 0.320	**0.003**	− 0.313	**0.004**

Adjusted for variables in basic and clinical parameters associated with blood pressure (age, sex, body mass index, current smoking status, and alcohol drinking status).

Night to day systolic blood pressure ratio (%) = (Daytime sysBP − Nighttime sysBP)/Daytime sysBP × 100.

Statistically significant values are in boldface.

*SBP* systolic blood pressure, *DBP* diastolic blood pressure.

^a^Based on linear regression analysis.

### Multivariate analysis

The results of a multivariate analysis of factors associated with macular ischemia are depicted in Tables 4 and 5. The multivariable linear regression analysis was adjusted for all parameters (age, sex, body mass index, current smoking status, and alcohol drinking status). The factor that remained significant in the multivariate analysis was the night-to-day systolic BP ratio (β = − 0.313, *p* = 0.004). In logistic regression analysis of macular ischemia and dipping patterns, the non-dipper group was 8.25 times riskier in multivariate analysis than the dipper group (*p* < 0.001, Table 5).

**Table 5:** Association between dipping pattern and macular ischemia using logistic regression analysis.

	Crude		Multivariate	
	OR	*p* value^a^	OR	*p* value^a^
Dipper	1		1	
Non-dipper	7.52 (2.69, 21.03)	** < 0.001**	8.25 (2.81, 24.21)	** < 0.001**

Adjusted for variables in basic and clinical parameters associated with blood pressure (age, sex, body mass index, current smoking status, and alcohol drinking status).

Night to day systolic blood pressure ratio (%) = (Daytime sysBP − Nighttime sysBP)/Daytime sysBP × 100.

Dipper ≥ 10%; Non-dipper < 10%.

Statistically significant values appear in boldface.

^a^Based on logistic regression analysis.

## Discussion

The correlation between the night-to-day systolic BP ratio and the degree of macular ischemia is notable. After adjusting for other factors, such as age, sex, body mass index, or current smoking and drinking status, we observed that the relationship between macular ischemia and night-to-day systolic BP ratio was statistically significant. This suggests that BRVO patients with non-dipping pattern are vulnerable to macular ischemia; therefore, they tend to have poor visual outcomes.

The association of RVO with systemic diseases, such as diabetes mellitus (DM), hypertension, hyperlipidemia, coronary artery disease, or hemorrhagic stroke, is well-known^7,20,21^. Particularly, hypertension has attracted attention as a systemic risk factor for RVO in Western European and Asian populations^22–24^. However, there are few studies related to the circadian variability of BP measured using 24-h ABPM, which can provide valuable information about a person's BP phenotype^17,25^. Systemic BP normally follows a circadian rhythm and decreases while sleeping. This is called a “dipper” pattern and is characterized by a decline of > 10% in average systemic BP from day to night.

In 2006, the Renin-Angiotensin System Study investigated the relationship between BP and the severity of diabetic retinopathy in normotensive individuals with type 1 DM but without clinical diabetic nephropathy^25^. Several recent studies have found that a non-dipping pattern is more frequently seen in patients with RVO than in normal control patients^17,26,27^. These observations suggest that non-dipping pattern is related to RVO. However, previous reports did not present any data on how macular changes varied according to the dipping patterns. Therefore, in this study, the authors investigated whether the 24-h ABPM patterns affect the development of macular ischemia, which is a critical factor for the visual outcomes in patients with BRVO. In our study, the univariate and multivariate analyses demonstrated that a non-dipping pattern of systolic BP is strongly associated with the development of macular ischemia in patients with BRVO.

The mechanism by which elevated BP at night leads to macular ischemia has not been determined yet. However, according to studies on capillaries of target organs, such as the kidney and cerebrum, a few mechanisms can be inferred. Oliveras et al. demonstrated that nocturnal hypertension is associated with an increase in microalbuminuria; this suggests that a continuously high BP that physiologically fails to fall during nighttime (dipping pattern) will ultimately lead to the destruction of a target organ’s capillary endothelium^27^. Neurological clinical studies have shown that cerebral microbleeds are associated with reverse nocturnal dipping in hypertensive patients with ischemic stroke^28^. Considering these studies, the capillary endothelium around the macula affected by nocturnal hypertension is likely to be more vulnerable to inflammation, edema, and ischemia caused by BRVO than the normal endothelium is. Macular ischemia caused by capillary dropout is a key factor of visual outcome in BRVO^9,28,29^. In this study, perifoveal capillary dropout was significantly more likely to occur in the non-dipper group than in the dipper group, leading to worse visual outcomes in the non-dipper group. Considering the associations between nocturnal hypertension and microvascular damage of various target organs, our results suggest that the constant occurrence of a non-dipping nocturnal pattern could lead to damage the capillary endothelial cells of the macula. This implies that the nocturnal decline in BP is a protective mechanism of the target organ’s capillaries. The absence of a nocturnal decline in BP could make the macula of patients with BRVO vulnerable to hypoxic conditions caused by venous obstruction.

Macular edema is also a cause of decreased vision in patients with BRVO. However, macular edema can be improved through treatment or resolved on its own^30,31^. Macular edema can be controlled early through intravitreal injection of anti-VEGF, which is recently developed effective treatment, although recurrence of macular edema has been frequently noted following anti-VEGF therapy^30–32^. Therefore, the long-term visual prognosis of patients with BRVO depends primarily on the presence or absence of macular ischemia^9,28,33,34^. In this study, macular edema was improved using anti-VEGF treatment in the non-dipper and dipper groups. Considering that the reduction in macular edema was greater in the non-dipper group than that in the dipper group, the former demonstrated a lower visual acuity during the final visit. If macular edema disappears after treatment, vision should improve; however, in the non-dipper group, even after the edema disappeared, not much vision improvement occurred, which can be attributed to macular ischemia. Therefore, as observed in our study, the night-to-day systolic BP ratio may be a risk factor for macular ischemia, thus affecting the visual outcome.

This study has several limitations. This study involved a relatively small number of patients (n = 86) and was designed retrospectively. Among the risk factors associated with BRVO, blood glucose and lipid levels related to blood tests could not be included due to data limitations. Further prospective studies are needed to measure BP at later stages in the course of the disease. Although the average values were measured through 24-h monitoring, there is a possibility of bias in the BP measurements. This is owing to variable activity patterns and sleep timings of individuals. In addition, patients with a history of hypertension or who have taken antihypertensive drugs were not included in this study, but patients with no symptoms due to latent hypertension may be included in this study. However, despite the limitations of this method, we believe that we proceeded with this study by carefully monitoring nocturnal BP in BRVO patients to identify patients with non-dipping BP patterns.

In conclusion, to the best of our knowledge, this is the first study to evaluate the prognostic factors for visual outcome in patients with BRVO by investigating the association between nocturnal BP pattern and macular ischemia. The evidence from this study shows that patients with BRVO who have non-dipping patterns might be vulnerable to macular ischemia, leading to poor visual outcomes. In our opinion, the 24-h ABPM method should be used to assess nocturnal BP patterns in patients with retinal vascular disease.

## Materials and methods

### Study design

This was a multicenter, retrospective study conducted at a tertiary medical center in Busan, South Korea. All data were collected and analyzed after approval of informed consent waived by the Institutional Review Board of Kosin University Gospel hospital (approval number: 2018-07-019), and the study adhered to the tenets of the Declaration of Helsinki. All experimental protocols were approved by the Institutional Review Board of Kosin University Gospel hospital. A medical chart review was performed to identify patients who were diagnosed with BRVO between January 2015 and December 2018. All included patients had BRVO and a macular layer thickness within the 6 mm diameter circle of the Early Treatment Diabetic Retinopathy Study (ETDRS) map. The exclusion criteria were as follows: diagnosis of glaucoma, vitreous hemorrhage, retinal arterial occlusion, or diabetic retinopathy and prior history of focal/grid or panretinal photocoagulation, macular disease, or intraocular surgery. All patients who had taken systemic medications for hypertension in the past were excluded. A thorough ophthalmic examination, including color fundus photography, optical coherence tomography (OCT), fundus fluorescein angiography (FFA), and 24-h ABPM, was conducted for all patients at the initial outpatient visit. The clinical characteristics of the patients, including age, sex, systemic medical history, visual acuity, IOP, axial length, and follow-up period, were acquired from medical charts. Best-corrected visual acuity based on spectacle correction was measured using a Snellen chart. It was then converted to the logarithm of the minimum angle-resolution visual acuity.

### Imaging protocol

Retina evaluation, including FFA and OCT, was performed for all patients using the Heidelberg Spectralis (Heidelberg Engineering, Heidelberg, Germany) platform. CMT and SFCT were measured by enhanced depth imaging (EDI)-OCT. CMT was recorded using an automated software present in the line scan pattern of OCT. The thickness values were defined as the foveal central subfield (1 mm from the center of the fovea).

SFCT was measured using the Heidelberg software caliper at a point under the fovea, from the base layer of Bruch’s membrane to the layer of the sclerochoroidal junction. Experienced reviewers who were blinded to the patients’ clinical data reviewed the choroidal thickness EDI-OCT scan data.

**Figure 2:**
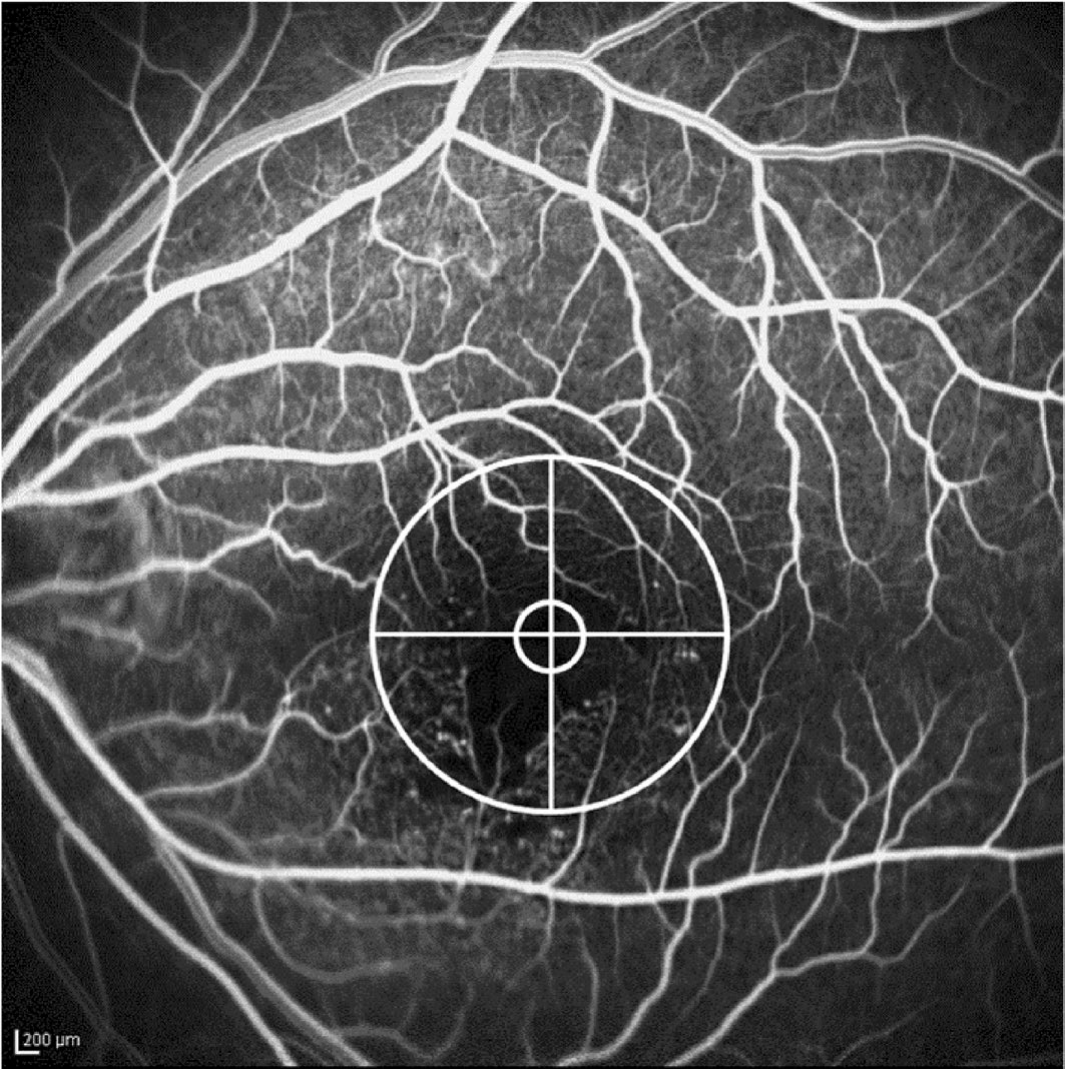
Quantitative assessment of capillary ring loss and the foveal avascular zone outline, which were measured using the 3 mm zone of the Early Treatment Diabetic Retinopathy Study grid circle. The image demonstrates the degree of macular ischemia in one quadrant.

Macular edema was defined as a radial cystic pattern in FFA images within a 3 mm diameter of the ETDRS grid. The degree of macular ischemia was assessed using FFA to evaluate macular perfusion state and foveal capillary ring loss, which was an outline of the foveal avascular zone (FAZ). And it was obtained in the initial outpatient visit. Patients with broken perifoveal capillary rings at the border of the FAZ, with a distinct area of capillary nonperfusion within the foveal center, were regarded as having macular ischemic BRVO. A quantitative assessment of the degree of macular ischemia was performed using the 3 mm zone of the ETDRS grid circle. The assessments were based on the four-quadrant area of capillary ring loss and FAZ outline breaks (Fig. 2).

### Ambulatory blood pressure monitoring

ABPM was performed for a full day (24 h). The BP of all patients with BRVO was measured at the initial outpatient visit with the same kind of instrument, and their data were analyzed using the same software (GE CardioSoft version 6.51; GE Medical Systems, Milwaukee, WI, USA). The monitoring device used in this study was programmed to measure BP at 30-min intervals from 6 AM to 10 PM (daytime) and at 60-min intervals from 10 PM to 6 AM (nighttime). When the BP values that exceeded certain limits (systolic BP ≥ 220 or ≤ 80 mmHg; diastolic BP ≥ 130 or ≤ 40 mmHg) were not recorded, the device repeated the measurements. All BP values were measured automatically (not manually measured).

Nocturnal hypertension was defined as nighttime systolic BP ≥ 120 mmHg or diastolic BP ≥ 70 mmHg^14^. The night-to-day systolic BP ratio was calculated as the ratio between the differences in mean daytime and nighttime systolic BP. The daytime systolic BP was calculated using the following formula: $\text{Night-to-day systolic} \, \text{BP ratio} \;\; (\%) = \frac{\text{Day sysBP-Night sysBP}}{\text{Day sysBP}}\times{ 100}$. Patients with a ratio of < 10% were defined as non-dippers^15^. For example, if a patient had a mean daytime systolic BP of 160 mmHg and a mean nighttime systolic BP of 140 mmHg, the mean ${\text{systolic}}\;{\text{BP}}\;{\text{dip}}\;{\text{would}}\;{\text{be}}\;\;\frac{{160\;{\text{mmHg}} - 140\;{\text{mmHg}}}}{{160\;{\text{mmHg}}}} \times 100\%  = 12.5\%.$ This patient would be considered to have a dipping pattern (and categorized as a dipper) owing to their ratio being above 10%. BRVO patients with dipping patterns were assigned to the dipper group, while those with non-dipping patterns were assigned to the non-dipper group.

### Statistical analysis

All patient data were collected using Microsoft Office Excel 2007 (Microsoft Corporation, Redmond, WA) and were entered into the electronic data-processing systems as mean ± standard deviation. Bivariate comparisons between the dipper and non-dipper groups were performed using a Student’s t-test for continuous variables and Pearson’s chi-square test for categorical variables. Univariate and multivariate analyses were performed to identify the factors associated with macular ischemia. All statistical analyses were conducted using SPSS software (version 18.0; IBM, Chicago, USA). Statistical significance was set at *P* < 0.05.
